# RUNX1 targeting AKT3 promotes alveolar hypercoagulation and fibrinolytic inhibition in LPS induced ARDS

**DOI:** 10.1186/s12931-024-02689-2

**Published:** 2024-01-24

**Authors:** Chuan Xiao, Jiaoyangzi Liu, Yumei Cheng, Yingxia Wu, Qing Li, Xianjun Chen, Jia Yuan, Qi Dong, Lu Li, Ying liu, Feng Shen

**Affiliations:** 1 Department of Intensive Care Unit, The Affiliated Hospital of Guizhou Medical University, Guiyang, China; 2 Department of Emergency, The Affiliated Hospital of Guizhou Medical University, Guiyang, China

**Keywords:** AKT3, Runt-related transcription factor 1, Alveolar hypercoagulation, Fibrinolytic inhibition, Acute respiratory distress syndrome

## Abstract

**Background:**

Alveolar hypercoagulation and fibrinolytic inhibition are mainly responsible for massive alveolar fibrin deposition, which are closely related with refractory hypoxemia in acute respiratory distress syndrome (ARDS). Our previous study testified runt-related transcription factor (RUNX1) participated in the regulation of this pathophysiology in this syndrome, but the mechanism is unknown. We speculate that screening the downstream genes associated with RUNX1 will presumably help uncover the mechanism of RUNX1.

**Methods:**

Genes associated with RUNX1 were screened by CHIP-seq, among which the target gene was verified by Dual Luciferase experiment. Then the efficacy of the target gene on alveolar hypercoagulation and fibrinolytic inhibition in LPS-induced ARDS was explored in vivo as well as in vitro. Finally, whether the regulatory effects of RUNX1 on alveolar hypercoagulation and fibrinolytic in ARDS would be related with the screened target gene was also sufficiently explored.

**Results:**

Among these screened genes, AKT3 was verified to be the direct target gene of RUNX1. Results showed that AKT3 was highly expressed either in lung tissues of LPS-induced rat ARDS or in LPS-treated alveolar epithelia cell type II (AECII). Tissue factor (TF) and plasminogen activator inhibitor 1 (PAI-1) were increasingly expressed both in lung tissues of ARDS and in LPS-induced AECII, which were all significantly attenuated by down-regulation of AKT3. Inhibition of AKT3 gene obviously ameliorated the LPS-induced lung injury as well as the collagen I expression in ARDS. RUNX1 overexpression not only promoted the expressions of TF, PAI-1, but also boosted AKT3 expression in vitro. More importantly, the efficacy of RUNX1 on TF, PAI-1 were all effectively reversed by down-regulation of AKT3 gene.

**Conclusion:**

AKT3 is an important target gene of RUNX1, through which RUNX1 exerted its regulatory role on alveolar hypercoagulation and fibrinolytic inhibition in LPS-induced ARDS. RUNX1/ATK3 signaling axis is expected to be a new target for the exploration of ARDS genesis and treatment.

**Supplementary Information:**

The online version contains supplementary material available at 10.1186/s12931-024-02689-2.

## Introduction

Acute respiratory distress syndrome (ARDS) is an acute inflammatory lung injury and one of the serious life-threatening forms of respiratory failure [1]. Refractory hypoxemia is an important pathophysiological feature of ARDS, which is a key factor associated with multiple organ dysfunction (MODS) and with the high mortality in ARDS [2–4]. In ARDS process, it has been seen a massive fibrin deposit in alveolar space, which contributes largely to the hypoxemia and the impaired lung compliance [5, 6].

Our previous studies have confirmed that alveolar hypercoagulation and fibrinolytic inhibition existed and are responsible for the fibrin deposit in alveoli [7, 8], ultimately resulting in refractory hypoxemia in ARDS.

It has been confirmed that alveolar epithelial cell type II (AECII) owns an important regulatory effect on alveolar hypercoagulation and fibrinolytic inhibition through expressing excessive tissue factor (TF) and plasminogen activator inhibitor 1 (PAI-1) in ARDS [9–11], but the mechanism is not clearly understood.

Runt-related transcription factor 1 (RUNX1) is a key transcriptional regulator involved in major developmental pathways including hematopoiesis, neurogenesis, and skeletogenesis [12]. Researches have clarified many significant functions of RUNX1 in hematopoietic development, hematopoietic stem cell homeostasis, and blood malignancies in the past 20 years [13]. What's more, in our previous experiment, RUNX1 was found to have the regulatory role in alveolar hypercoagulation and fibrinolytic inhibition in LPS-induced ARDS [14, 15], but its underlying mechanism still remains to be further explored. We hypothesize that some target genes might exist through which RUNX1 promotes AECII cell to excessively express TF and PAI-1 in ARDS.

In this experiment, genes associated with RUNX1 were screened by CHIP-seq, among which the AKT3 was verified by dual Luciferase to be a target gene of RUNX1. Then the efficacy of AKT3 on alveolar hypercoagulation and fibrinolytic inhibition, and the relationship between RUNX1 and AKT3 in regulating alveolar hypercoagulation and fibrinolytic inhibition in LPS-induced ARDS were all thoroughly studied. Meanwhile, AKT3 associated phosphatidylinositol-3-kinase (PI3K) / AKT signaling pathway was also preliminarily observed.

## Methods and materials

### Experimentin vitro

#### Cell culture and LPS stimulation

Since AECII cell has been confirmed to be the main effector cell in regulating alveolar hypercoagulation and fibrinolytic inhibition in ARDS, RLE-6TN cell (AECII of rat) was used to be the experimental subject. RLE-6TN was purchased from Cell Center, Xiangya School of Medicine, Central South University. The cells was cultivated in 37 °C and 5% CO_2_ environment. The culture medium consisted of 10% fetal bovine serum (FBS) + Duchenne modified eagle medium (DMEM, Gibco, USA). Replication of LPS-induced cell injury model or control for AECIIs: LPS (5 mg/L) or the same dose of PBS incubated for 24 h. Cells were randomly treated either with LPS (5 mg/L) or with a same volume of PBS for 24 h. In addition, the cells was induced with LPS for several period of time including 24 h so that the dynamic changes of target gene in cells could be observed.

#### Chromatin immunoprecipitation-sequencing (CHIP-Seq)

In order to find out the downstream target genes of RUNX1, the CHIP-seq was performed. ChIP-Seq service was provided by CloudSeq Biotech (Shanghai, China). Chromatin Immunoprecipitation was performed with GenSeq® Chromatin Immunoprecipitation (ChIP) Kit (GenSeq Inc.) according to the manufacturer’s instructions. The yield of ChIPed DNA was determined via Quant IT fluorescence assay (ThermoFisher), and sequencing libraries were generated with GenSeq® Rapid DNA Library Prep Kit (GenSeq Inc.) by following the manufacturer’s manual. The library quality was determined by using Agilent 2100 Bioanalyzer (Agilent), and then, subjected to high-throughput 150 base paired-end sequencing on Illumina NovaSeq sequencer. Briefly, raw data were generated after sequencing, image analysis, base calling and quality filtering on Illumina Novaseq6000 sequencer. Firstly, Q30 was used to perform quality control. After adaptor-trimming and low-quality reads removing by cutadapt (v1.9.3) software, high quality clean reads were generated. Then these clean reads were aligned to reference genome using bowtie 2 software (v2.2.4) with default parameters. Peak calling was performed with MACS software (v2.2.7.1). Differentially enriched regions were identified by diffReps software (v1.55.4). The enriched peaks were then annotated with the latest UCSC RefSeq database to connect the peak information with the gene annotation. The enriched peaks were visualized in UCSC Genome Browse.

#### Luciferase reporter assay

To validate the target binding of the screened gene to RUNX1, dual luciferase assay was conducted. HEK-293 cells were plated in a 24-well plate overnight, transfection was performed at about 70% cell coverage. Next day, cells were co-transfected with the control pcDNA3.1 empty vector (EV, 250 ng) or the wild-type RUNX1 plasmid (250 ng)-based firefly luciferase reporter plus the renilla luciferase control vector pRL-TK (25 ng) using the HG transgene reagent (genomeditec, TG-10012-S). After transfection (48 h later), cell lysates were prepared and firefly/renilla luciferase values were quantified using Firefly Luciferase Assay (Genomeditech). Firefly luciferase values were normalized to Renilla luciferase values and expressed as relative values.

#### Cell lines construction with RUNX1 overexpression and target gene down-expression

To construct cell lines with RUNX1 overexpression, lentiviral vector with RUNX1 overexpression was generated and transfected into RLE-6TN. After transfection, cells was screened with puromycin and termed LV- RUNX1. Moreover, the lentiviral system with expression cassette was set as control. Lentiviral construction and transduction of RUNX1 were performed by Tsingke Biotechnology (Beijing, China). The oligonucleotide sequences of lentiviruses were listed in Additional file 2: Table S1.

To down-regulate the target gene, siRNA transfection was used with Lipofectamine RNAiMAX (Invitrogen) on 10–40 nM of siRNA. Additional experiments were completed at least 48 h after siRNA transfection. More information on siRNA sequences was seen in Additional file 2: Table S2.

#### RNA extraction, quantitative real-time PCR (qPCR)

qPCR was performed to determine the mRNA expression of target gene, TF, and PAI-1. Total RNA in RLE-6TN was extracted by using TRIzol reagent (Invitrogen), followed by cDNA synthesis, where the cDNA synthesis mix was purchased from Takara, and cDNA was diluted and amplified according to the manufacturer’s instructions and was measured by using a qPCR system. The relative expression of the target gene was calculated by the 2^−ΔΔCt^ method. Amplified transcript levels for each specific gene was normalized to GAPDH. Primers were provided by Sacred Engineering, see Additional file 2:Table S3.

#### Western blots (WB)

For the identification and semi-quantitative analysis of AKT3, TF, PAI-1 and phosphorylated (p)-AKT protein, WB was performed with the total protein mixture. The total protein lysate was extracted by SDS–PAGE and transferred into PVDF membranes and was then incubated with primary antibodies and secondary antibodies in sequence followed by exposure. Anti-RUNX1 antibody (1:2000), anti-PAI-1 antibody (1.5:10,000), anti-p-AKT antibody (1:3000), anti-AKT3 antibody (1:500) and anti-GAPDH antibody (1:2000) were purchased from Wuhan Sanying Biotechnology Co. Anti-TF antibody (1:2000) was obtained from NOVUS. Secondary antibodies were anti-rabbit or anti-mouse IgG were purchased from Solarbio, China.

### Experimentin vivo

#### Animals

Male Sprague–Dawley rats (180–250 g) were purchased from Beijing Weitong Lihua Laboratory Animal Technology Co Ltd (Beijing, China) (Certificate No. 2021116Aazz0619000338) and all were housed in a specific pathogen-free (SPF) laboratory at 25 ± 2 °C with a 50/50 split between daily light and dark hours.

#### ARDS model replication

The rats were anesthetized with isoflurane (2–3%), and then were inhaled LPS (10 mg/kg, Beijing Solebo Technology Co. LTD) through the nebulizer Spray. Rats in control were nebulized with the same volume of normal saline as LPS. Twenty-four hours after LPS inhalation, rats were anesthetized and bled to death. All procedures of the experiment were approved by the Institutional Animal Care and Use Committee of Guizhou Medical University.

#### AKT3 down-expression in lung tissue

To illustrate the effect of AKT3 on alveolar hypercoagulation and fibrinolysis inhibition in vivo, si-AKT3 was designed and synthesized by Sangon Biotech (Shanghai, China). si-AKT3 and scrambled siRNA control (SCR) were inhaled into the lungs.

#### RNA extraction, quantitative real-time PCR (qPCR)

qPCR was performed to determine the mRNA expression of AKT3, TF, and PAI-1. Total RNA in the lung of Rats was extracted by using TRIzol reagent (Invitrogen). The remaining steps are the same as in the cell experiment.

#### Western blots (WB)

For the identification and semi-quantitative analysis of AKT3, TF, and PAI-1, WB was performed with the total protein mixture. The tissue should be shredded as much as possible, and 1 mL of lysate (80 μL RIPA + 10 μL protease inhibitor + 10 μL phosphatase inhibitor + 1 μL PMSF) should be added for every 100 mg of lung tissue for protein extraction. The remaining steps are the same as in the cell experiment.

#### Wet-to-dry ratio

To assess the severity of pulmonary edema, wet/dry weight ratio of lung tissue was measured. Chest cavity of rat was carefully opened and the left lung tissue was removed, being rinsed with pre-cooled sterile water. After the residual blood on the lung surface was thoroughly removed, the lung tissue was weighed and recorded as wet weight (W), and then baked in a constant temperature oven (80℃) until the lung tissue weight no longer changes (about 72 h), of which the weight at this point was regarded as the dry weight (D) of left lung tissue. Wet-to-dry ratio (W/D) was then calculated.

#### Hematoxylin and eosin staining

Pathological changes in the lung tissue were performed by hematoxylin and eosin (HE) staining. The right lungs were fixed (4% paraformaldehyde solution). HE images were taken by light microscopy (Leica, Germany) at a magnification of × 200. Lung injury scores (LIS) were assessed within three random regions of lung tissue by using five independent variables according to the Matute-Bello and Diffuse Alveolar Damage (DAD) method [13] as follows:

**Table:** 

Variables	Ratings
(A)	Neutrophils in the alveolar space (none = 0, 1 to 5 cells = 1, > 5 cells = 2)
(B)	Neutrophils in the interstitial space (none = 0, 1 to 5 cells = 1, > 5 cells = 2)
(C)	Hyaline membranes (none = 0, one membrane = 1, greater than one membrane = 2)
(D)	Protein debris in the air spaces (none = 0, one case = 1, more than one case = 2)
(E)	Alveolar septal thickening (< 2 × simulated thickness = 0, 2–4 × simulated thickness = 1, > 4 × simulated thickness = 2)
LIS	[(20 × A) + (14 × B) + (7 × C) + (7 × D) + (2 × E)]/100

#### Immunohistochemistry

Collagen I levels were determined by immunohistochemistry [14]. Collagen I antibody [EPR24331-53, Britain, (ab270993)] and a rabbit two-step kit (PV-6001) were purchased from Origene (China). The staining result was the total multiplication of the staining intensity score and the percentage of positive cells. The intensity of staining was scored as follows: 0, negative; 1, weak; 2, moderate; 3, strong. The number of positive cells is defined as follows: 0, < 5%; 1, 6–25%; 2, 26–50%; 3, 51–100%.

### Statistical analysis

The data were analyzed by GraphPad Prism 9.0 software and the quantitative data were fed in mean ± standard deviation (x ± s). The row indicates that differences between the two groups were analyzed using the t-test, and one-way variance was used for comparisons between more than two groups divide. *P* < 0.05 is considered to be statistically significant.

## Results

### Results in vitro

#### AKT3 is a direct transcriptional target of RUNX1

**Fig. 1:**
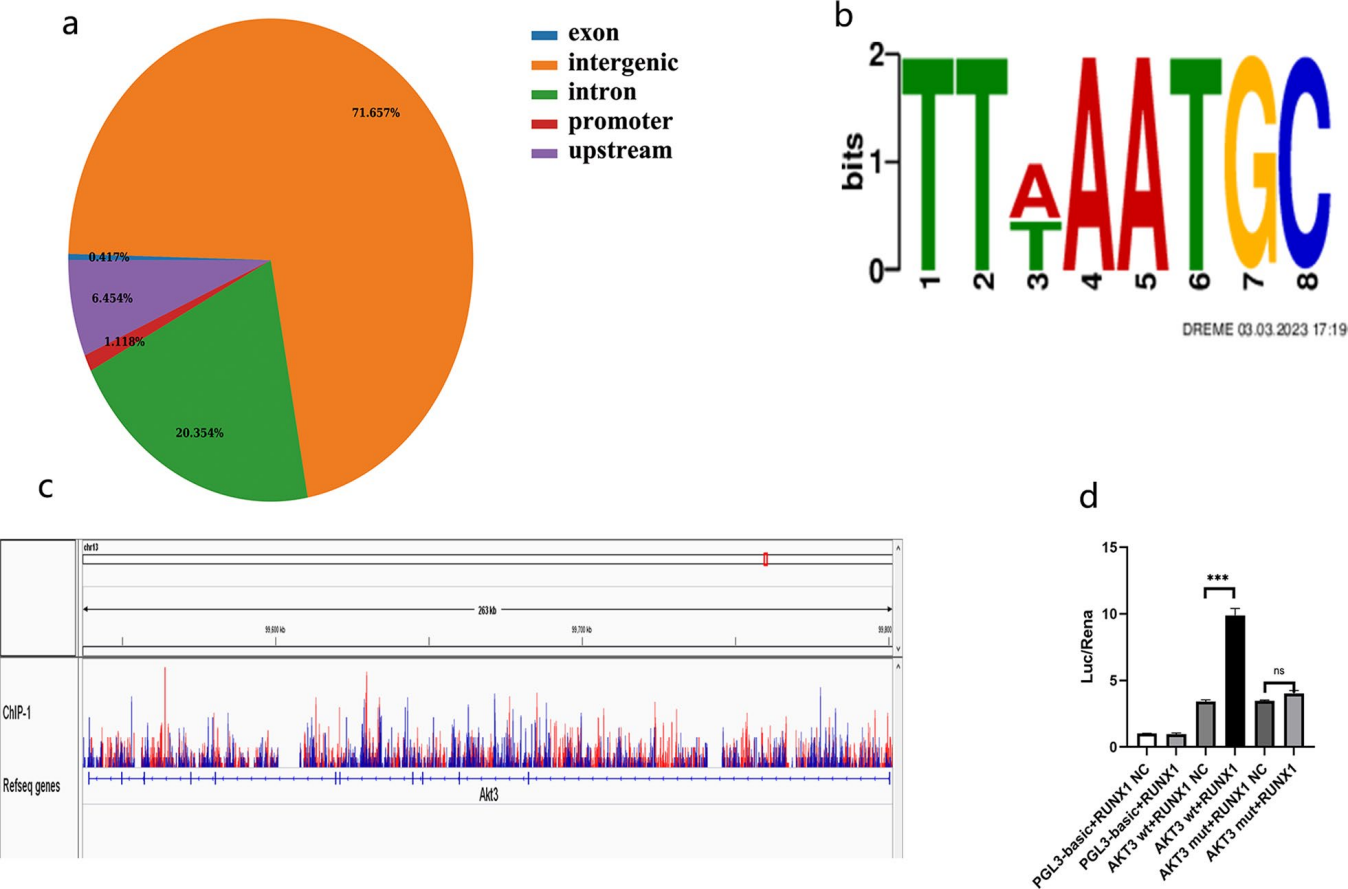
RUNX1 targets AKT3 expression. ChIP-seq analysis is performed using flag-tagged RUNX1 in AECII treated with LV-RUNX1 (**a**–**c**). Pie chart depicting the genomic distribution of RUNX1-enrichment (**a**). Motif analysis of the RUNX1 bound regions. Genome browser view showing RUNX1 ChIP-seq signal around AKT3 (**b**). RUNX1 peak in the promoter region on AKT3 (**c**). Activity of luciferase with cloned the full-length AKT3 promoter into a luciferase reporter plasmid (n = 3) (**d**). Values are presented as mean ± SD. ^ns^*P* > 0.05 and ***P < 0.001. *LV*  Lentiviral vectors, *NC*  Negative control

To explore the downstream signaling of RUNX1, a Chromatin Immunoprecipitation Sequencing (ChIP-seq) assay was conducted by using RUNX1 antibody in RUNX1-overexpressed RLE-6TN cells subjected to LPS stimulation. Thousands of differential RUNX1-bound chromatin regions were identified by using ChIP-seq (Fig. 1a). Motif discovery using these RUNX1-bound regions yielded the consensus. RUNX1 motif provided validation for the ChIP-seq dataset (Fig. 1b). For the number of RUNX1-mediated genes to be narrowed, genes associated with RUNX1-bound promoter region and genes with ARDS fibrosis regulatory function were screened. Among these screened genes, AKT3 was found to have a significant RUNX1-binding promoter region (Fig. 1c). A direct interaction between RUNX1 and AKT3 gene promoter element was validated by dual luciferase assay. A full-length (FL) AKT3 promoter was cloned into a luciferase reporter plasmid. Data showed that AKT3 promoter activity was significantly elevated in the presence of RUNX1 overexpression (Fig. 1d). These results supported the notion that RUNX1 directly regulates AKT3 transcription.

#### Changes in AKT3 expression in LPS-induced RLE-6TN cells

**Fig. 2:**
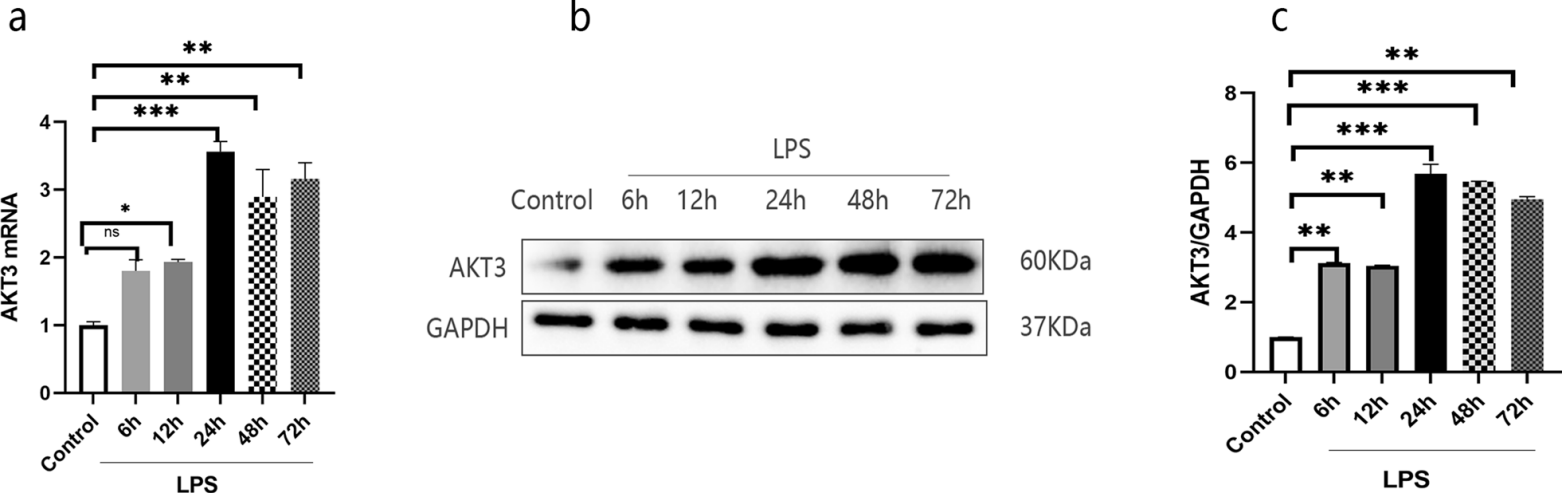
AKT3 expression in LPS-induced AECII cells. The expression of AKT3 mRNA (**a**), AKT3 protein (**b**) and the histogram of semiquantitative analysis of AKT3 protein (**c**) are displayed. Values are presented as the mean ± SD (n = 3). ^ns^ > 0.05, *P < 0.05, **P < 0.01 and ***P < 0.001; *LPS*  Lipopolysaccharide, *AECII* alveolar epithelial cells type II

In our experiment in vitro, the dynamic expression of AKT3 in LPS-treated RLE-6TN cells was observed. As shown in Fig. 2, the expression of AKT3 in mRNA and in protein gradually increased, reaching a peak at 24 h point and then a slight decrease at 48 and 72 h point of LPS stimulation. The results showed that the dynamic changes of AKT3 under LPS stimulation exhibited a time dependence.

#### AKT3 promotes the expression of TF and PAI-1 in LPS-induced AECII cells

**Fig. 3:**
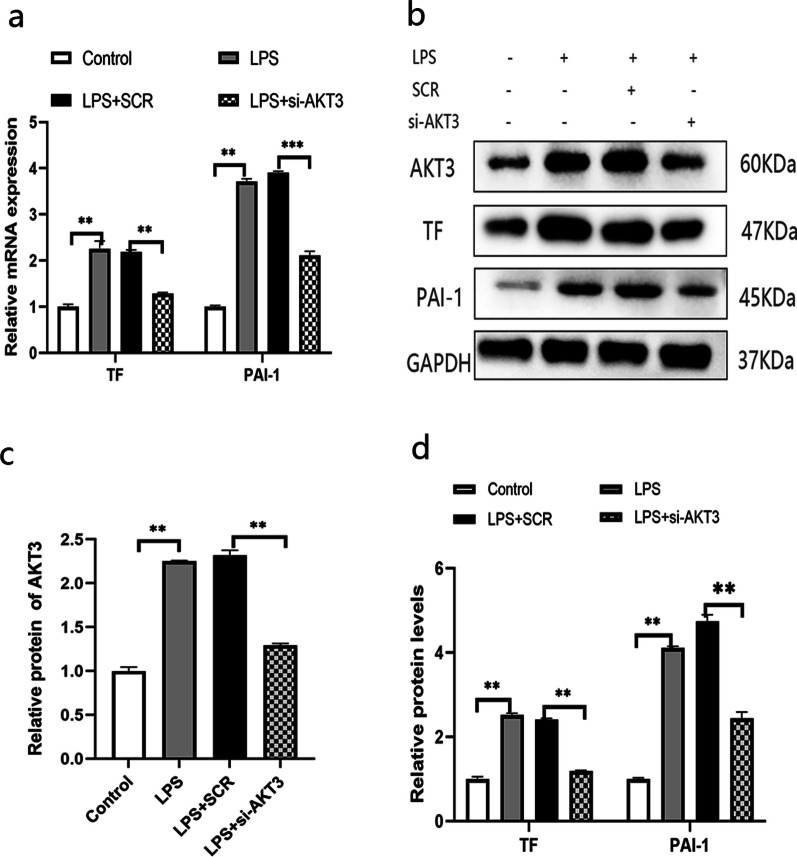
Impact of AKT3 on the expressions of TF and PAI-1 in LPS-induced AECII cells; TF and PAI-1 mRNA is analyzed by qPCR analysis (**a**) and their protein by Western Blot (**b**). Histogram of semiquantitative analysis of WB (**c**, **d**) are presented. **P < 0.01, ***P < 0.001. The values presented are the mean ± SD. (n = 3). *SCR*  scrambled siRNA control, *LPS*  Lipopolysaccharide, *AECII* alveolar epithelial cells type II. The samples are derived from the same experiment and that gels/blots are processed in parallel

To examine whether AKT3 regulates those factors in cell associated with alveolar hypercoagulant and fibrinolytic inhibition in LPS-induced ARDS, we down-regulated AKT3 through siRNA transfection of cells. Results demonstrated that AKT3 expression was effectively reduced by si-AKT3 #1, si-AKT3 #2 and si-AKT3 #3, with a maximal decrease of more than 50% in si-AKT3 (Additional file 1: Fig. S1). Therefore, si-AKT3 #3 was chosen for the subsequent experiments. Experimental data indicated that si-AKT3 dramatically attenuated the effects of LPS on those factors associated with alveolar hypercoagulation and fibrinolytic inhibition in cell (Fig. 3a–d).

#### RUNX1 positively regulates AKT3 and pI3K/Akt signaling pathway

**Fig. 4:**
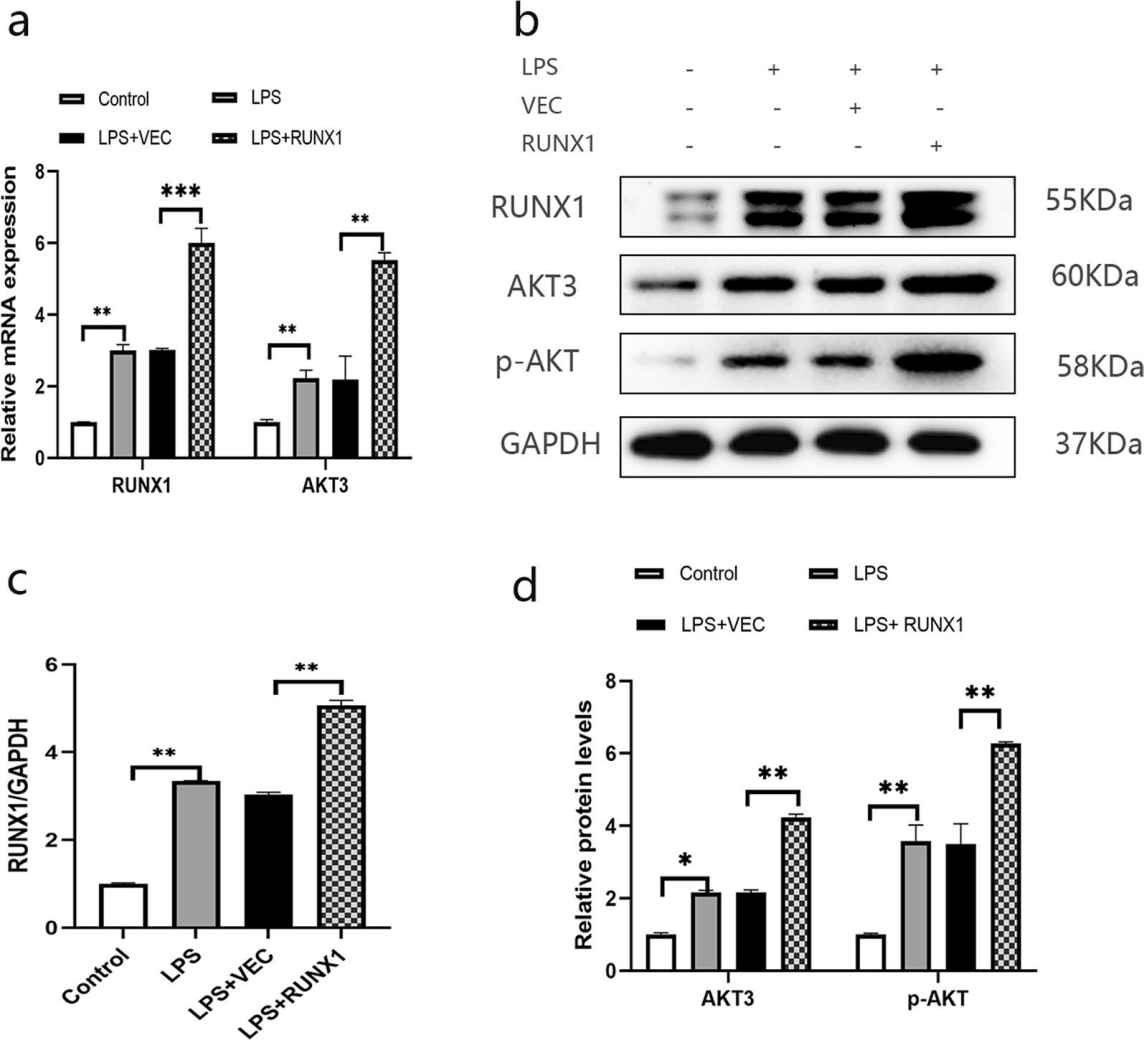
RUNX1 regulates the AKT3 and pI3K/Akt Signaling Pathway in LPS- induced AECII. RUNX1 and AKT3 mRNA are analyzed by qPCR analysis (**a**) and expressions of RUNX1, AKT3 and phosphorylated AKT (p-AKT) are analyzed by Western Blot (**b**). Histogram of semiquantitative analysis for proteins are presented (**c**, **d**); Values are presented as mean ± SD (n = 3). *P < 0.05, ** *P* < 0.01 and ***P < 0.001; *LPS*  Lipopolysaccharide, *LPS*  Lipopolysaccharide, *LV*  lentivirus, *AECII* alveolar epithelial cells type II. The samples are derived from the same experiment and the gels/blots are processed in parallel

In order to testify whether RUNX1 regulates AKT3 expression, RUNX1 gene was overexpressed (Fig. 4a–c). Results displayed that up-regulation of RUNX1 significantly boosted AKT3 expression in mRNA and in protein (Fig. 4a, b, d).

In addition, given that AKT3 is one of the important downstream molecules of the PI3K/AKT signaling pathway, so we also simultaneously observed the expression of phosphorylated (p-) AKT which stands for the activated status of the signaling pathway. Data showed that LPS treatment increased p-ATK expression in mRNA as well as in protein, which were obviously enhanced by RUNX1 overexpression (Fig. 4b, d), indicating that RUNX1 also activates PI3K/AKT signaling pathway in condition of LPS stimulation.

#### Downregulation of AKT3 rescues the regulatory RUNX1 effect on those factors associated with alveolar hypercoagulation, fibrinolysis inhibition and pI3K/Akt signaling pathway

**Fig. 5:**
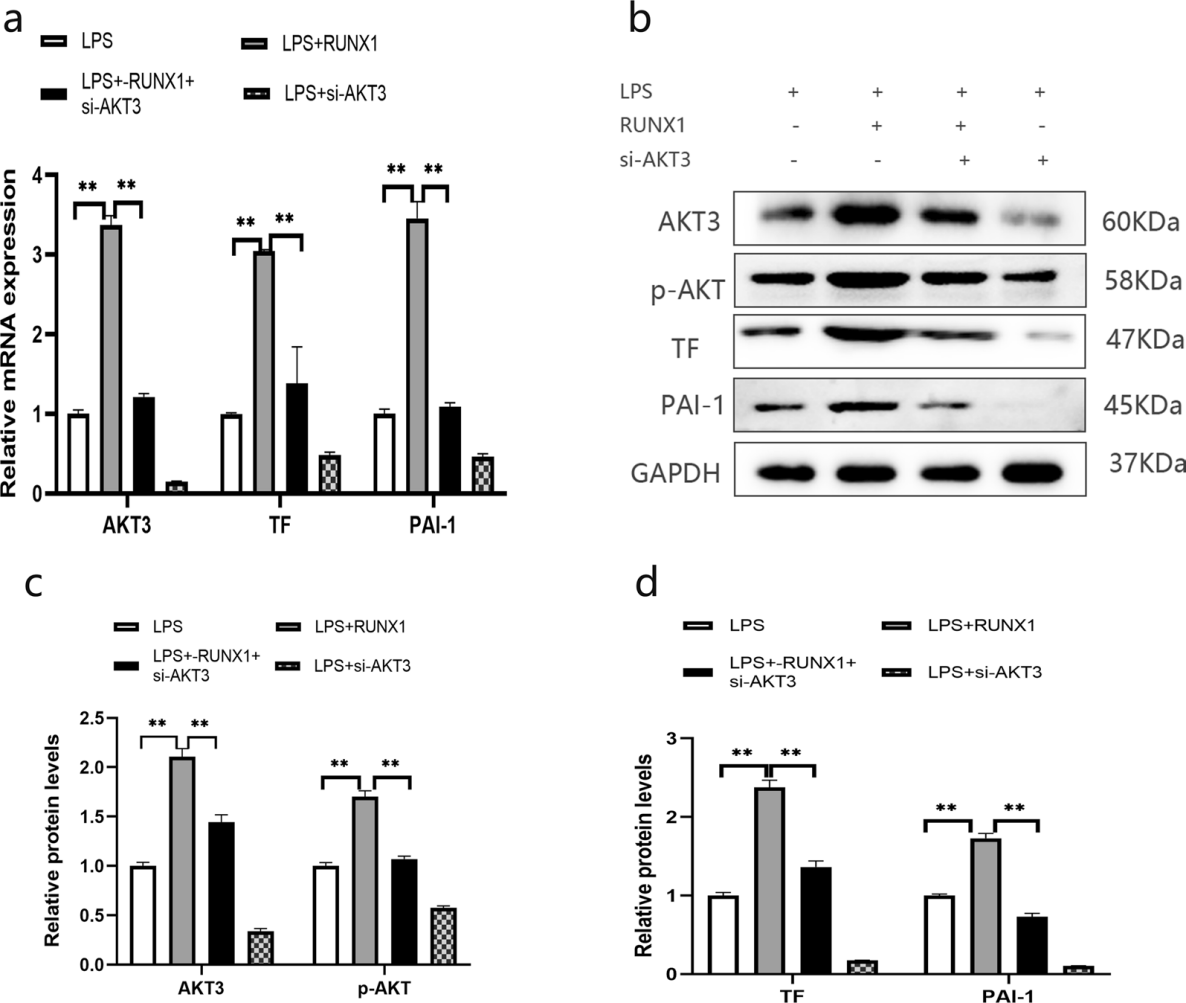
AKT3 rescues the effect of RUNX1 on alveolar hypercoagulation and fibrinolysis inhibition in LPS-induced AECII. All the indicators from the LPS-induced AECII which transfected with LV-RUNX1,si-AKT3, or both of them. mRNA levels of AKT3, TF and PAI-1 (**a**). protein level of AKT3, phosphorylated AKT (p-AKT), TF, and PAI-1 in AECII (**b**). protein semiquantitative analysis for AKT3, p-AKT, and TF, PAI-1. Values are presented as mean ± SD. (n = 3) (**c**, **d**). **P < 0.01. *LPS*  Lipopolysaccharide, *LV*  lentivirus, *AECII* alveolar epithelial cells type II. The samples are derived from the same experiment and that gels/blots are processed in parallel

In order to explore whether RUNX1 exerts its regulatory role through AKT3 or not, a rescue experiment was performed, with RUNX1 up-regulation accompanied with AKT3 down-regulation in cells (Fig. 5a–c). Experimental data displayed that the LPS-induced TF and PAI-1 expressions were significantly enhanced by RUNX1 up-regulation. However, the efficacy of RUNX1 up-regulation was effectively reversed by the concomitant downregulation of AKT3 (Fig. 5a, b, d), indicating that through AKT3 is one of the mechanisms of RUNX1 in regulating TF and PAI-1 in LPS-stimulated AECII. Meanwhile, the effect of RUNX1 up-regulation on PI3K/AKT pathway was also reversed by AKT3 down-regulation (Fig. 5b, c).

### Results in vivo

#### AKT3 aggravates lung injury in LPS-induced ARDS rats

**Fig. 6:**
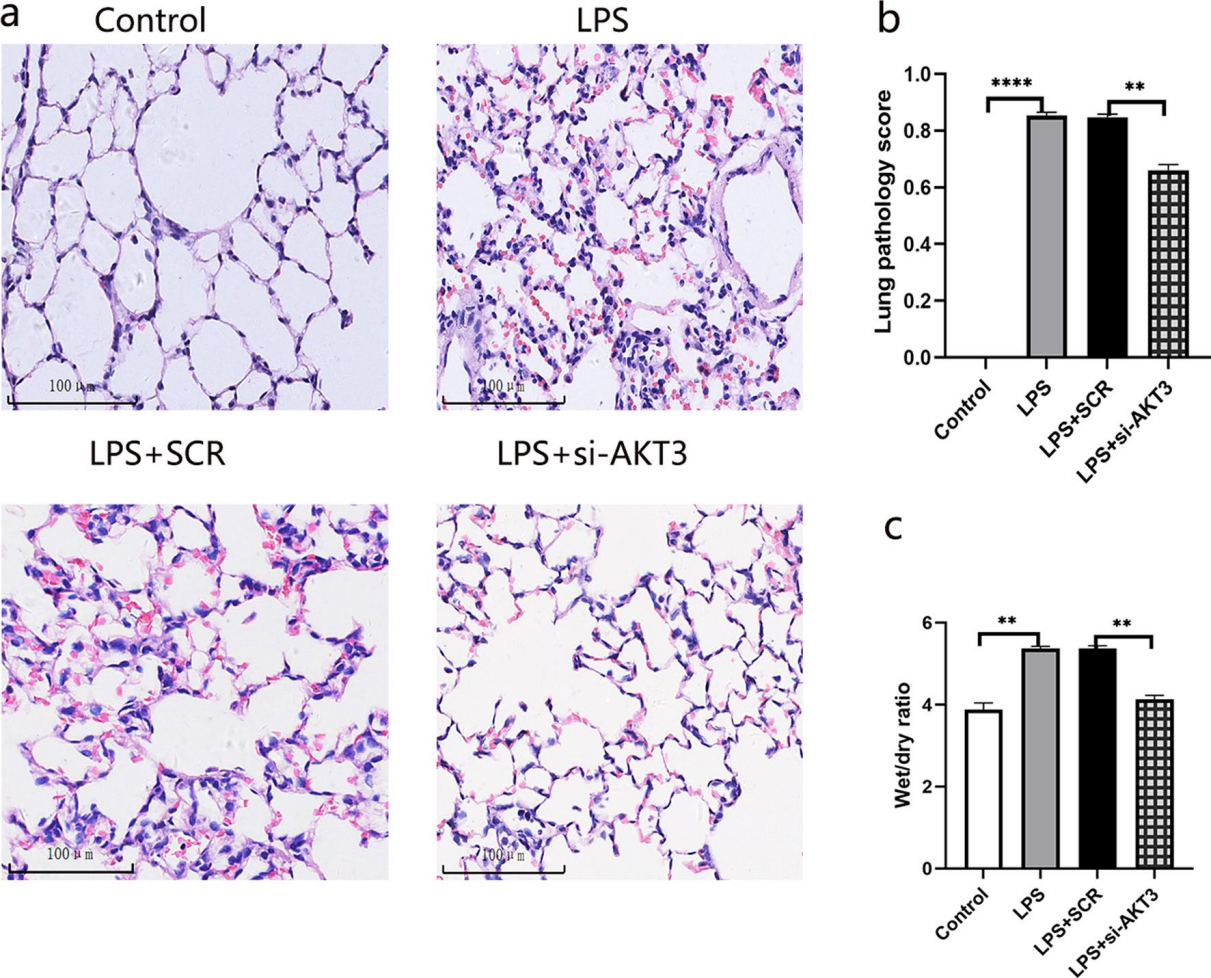
AKT3 is associated with lung injury in LPS-induced ARDS rats. Pathological changes in lung tissue, HE Scale bars, 100 um (n = 6) (**a**); the lung injury score (**b**); wet/dry (W/D) ratio (**c**). Values are presented as mean ± SD. **P < 0.01 and ****P < 0.0001. *LPS*  Lipopolysaccharide. *ARDS*  acute respiratory distress syndrome. *SCR*  scrambled siRNA control; *HE*  hematoxylin and eosin staining. *LPS*  Lipopolysaccharide

**Fig. 7:**
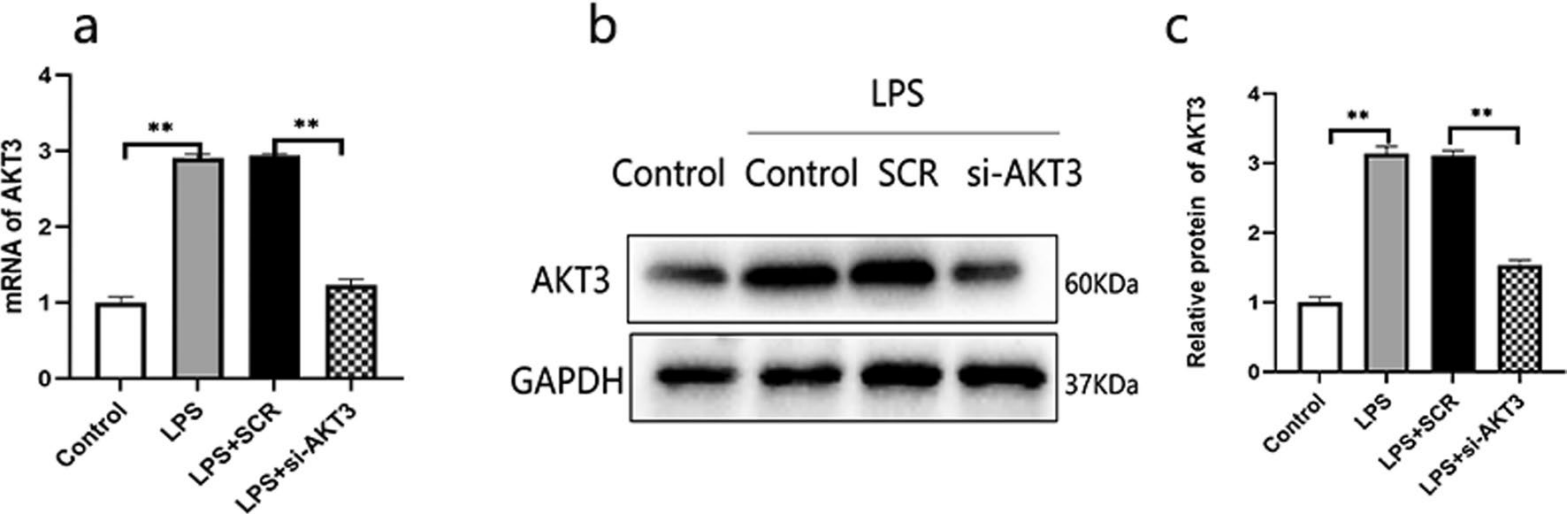
The expression of AKT3 in LPS-induced ARDS rats. Expression of AKT3 in mRNA and in protein were analyzed by qPCR analysis (**a**) and by WB (**b**) respectively. Protein semiquantitative analysis for AKT3 (**c**). Values are presented as mean ± SD (n = 6). **P < 0.01. *SCR*  scrambled siRNA control, *LPS*  Lipopolysaccharide, *ARDS*  acute respiratory distress syndrome. The samples are derived from the same experiment and that gels/blots are processed in parallel

In ARDS rat, it was seen a higher lung injury score, increased W/D ratio of lung tissue and an obvious intra-alveolar exudation, indicating acute lung injury (Fig. 6). AKT3 expression was increased in ARDS pulmonary tissue (Fig. 7).

In order to explore whether AKT3 aggravates lung injury, AKT3 gene in pulmonary tissue was successfully down-regulated through AKT3-siRNA nebulization (Fig. 7). Results indicated that AKT3 down-regulation significantly attenuated LPS-induced lung injury, indicated by those ameliorations in W/D ratio, intra-alveolar exudation and lung injury score (Fig. 6).

#### AKT3 promotes the expression of collagen I in ARDS rats

**Fig. 8:**
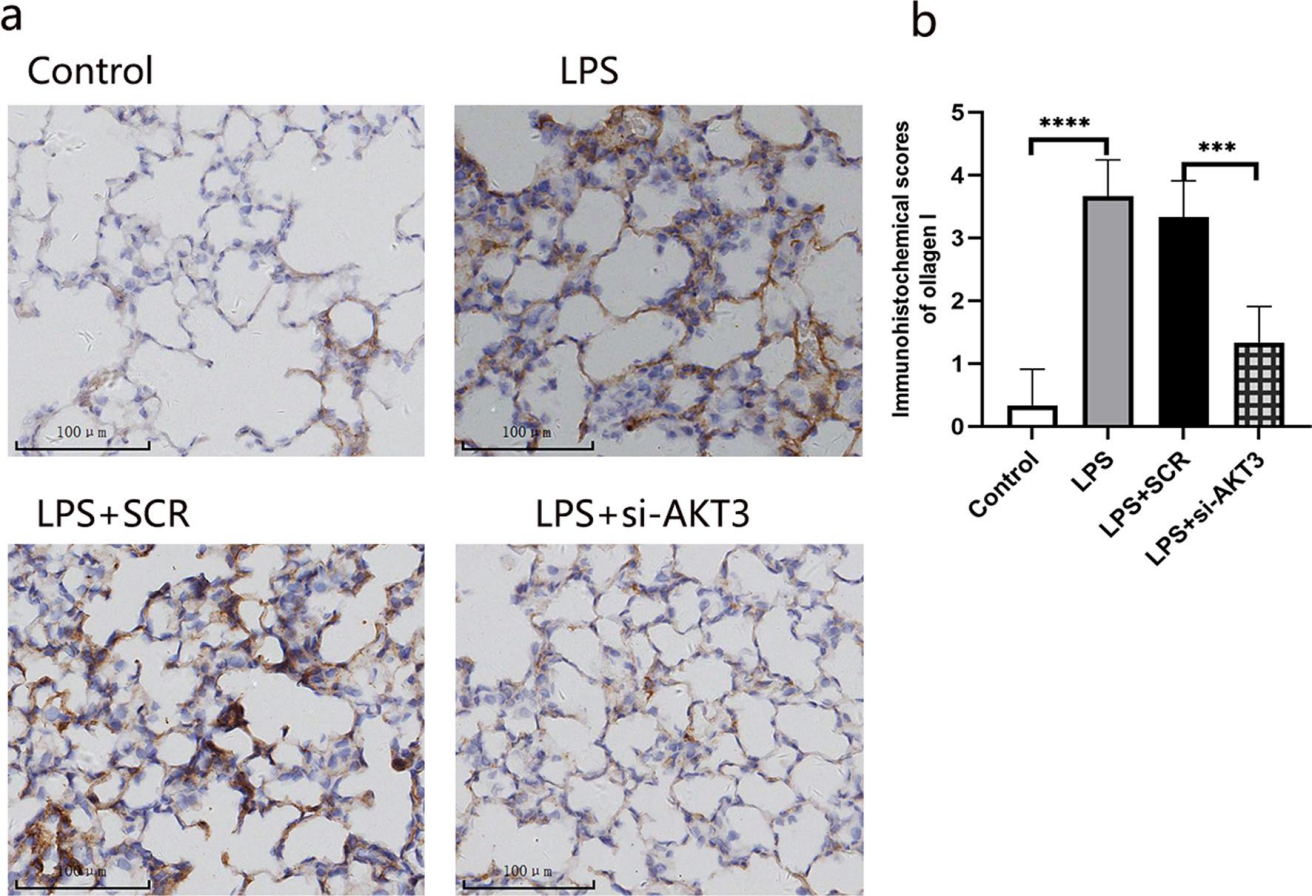
The expression of collagen I in lung tissue. collagen I expression in lung tissue (**a**). immunohistochemical scores of collagen I (**b**). Scale bars, 100 um (n = 6), magnification view (× 200). Values are presented as mean ± SD. ***P < 0.001 and ****P < 0.0001. *SCR*  scrambled siRNA control, *LPS*  Lipopolysaccharide

To explore the effect of AKT3 on fiber content in pulmonary tissue, collagen I level was determined by using immunohistochemistry histochemical assay. Results showed that lung tissue Collagen I level in ARDS rat was significantly elevated, but in condition of AKT3 down-regulation, however, collagen I level sharply decreased, even lower than that with LPS stimulation only (Fig. 8). Our data demonstrated that the increase in collagen I induced by LPS is mediated by AKT3.

#### AKT3 boosts the expressions of TF and PAI-1 in lung tissue of ARDS rats

**Fig. 9:**
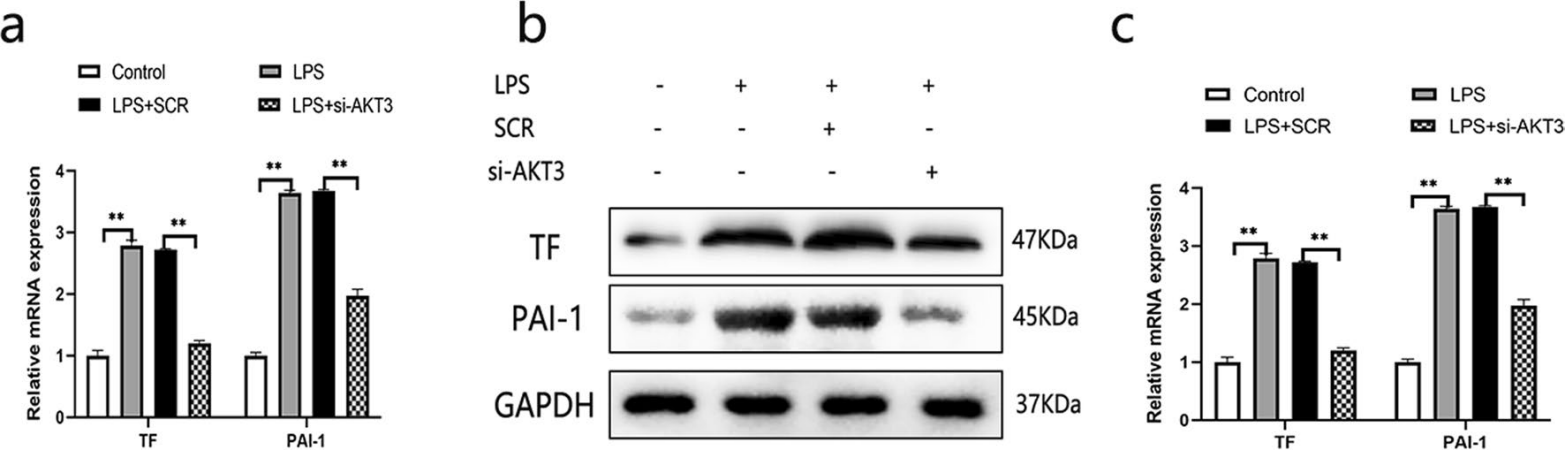
Impact of AKT3 on the expression of TF and PAI-1 in lung tissue. TF and PAI-1 mRNA (**a**). protein expression of TF, PAI-1 (n = 6) (**b**). Protein semiquantitative analysis for TF, PAI-1 (**c**). The values are presented the mean ± SD. **P < 0.01. *SCR*  scrambled siRNA control, *LPS*  Lipopolysaccharide. The samples derive from the same experiment and that gels/blots were processed in parallel

The effects of AKT3 on TF and PAI-1 in lung tissue were also investigated, helping to decide whether AKT3 regulates alveolar hypercoagulation and fibrinolytic inhibition in ARDS. LPS inhalation resulted in an increase in TF and PAI-1, either in mRNA or in protein, which are consistent with our previous data. Down-regulation of AKT3 markedly weakened TF and PAI-1 expression in ARDS rats (Fig. 9), suggesting that LPS induced alveolar hypercoagulation and fibrinolytic inhibition at least is partly mediated through AKT3.

## Discussion

In our previous study, RUNX1 was confirmed to participate in the regulation of alveolar hypercoagulation and fibrinolytic inhibition in ARDS. Therefore, exploring the mechanism of RUNX1 is beneficial for revealing the pathogenesis of ARDS. In our experiment, we are trying to find out the downstream signaling molecules of RUNX1. By means of ChIP-seq, we screened a series of genes associated with RUNX1 in LPS-stimulated AECII cells. By focusing on screening genes associated with the RUNX1-binding promoter region and genes with the ability to promote ARDS-associated pulmonary fibrosis, we successfully found out that AKT3 is a downstream target gene of RUNX1. Through luciferase reporter assay, we found that RUNX1 transfection significantly increased the luciferase activity of AKT3, and RUNX1 boosted the transcription and translation of AKT3. Therefore, we have the reason to think that AKT3 is a direct downstream target of RUNX1.

First, we dynamically observed the expression of AKT3 in AECII cell under LPS stimulation. After 6 h of LPS induction, AKT3 expression began to increase, reached its maximal level at 24 h, and then a slight decrease at 48 h and at 72 h, but all were significantly higher than in control cell. Based on the dynamic changing trajectory of AKT3, we treated cells with LPS for 24 h in the subsequent cell experiment.

Next, we explored the effects of AKT3 on TF and PAI-1, the two important factors responsible for alveolar hypercoagulation (TF) and fibrinolytic inhibition (PAI-1) respectively. In experiment in vitro, we found that LPS stimulation caused excessive expression of TF and PAI-1, but the effects of LPS was dramatically attenuated by AKT3 down-regulation. In ARDS rat, we discovered that AKT3 highly expressed in lung tissue, and interestingly, silencing AKT3 gene significantly weakened TF and PAI-1 expression induced by LPS, and the LPS-induced high level of collagen I was also decreased in condition of AKT3 down-regulation. All the results implied that AKT3 regulated the alveolar hypercoagulation and fibrinolytic inhibition in ARDS. Our findings are similar to those of other studies, in which AKT3 promoted the formation of arterial thrombosis in vivo by inactivating glycogen synthase-3 induced by thrombin [16, 17]. Meanwhile, in our experiment, AKT3 was also found to be associated with LPS-induced lung injury, proven by the amelioration of W/D ratio, intra-alveolar exudation and lung injury score when AKT3 was conditionally low-expressed.

AS the core content of our study, the relationship between RUNX and AKT3 was examed. First, we conditionally up-regulated RUNX1 gene through transfection with lentiviral vector containing RUNX1 overexpression. Results indicated RUNX1 overexpression dramatically boosted AKT3 level as well as TF and PAI-1 expressions in LPS-induced cells, and AKT3 up-regulation largely rescued the effects of RUNX1 on TF and PAI-1. The data suggested that RUNX1 regulates alveolar hypercoagulation and fibrinolytic inhibition by targeting AKT3.

At last, since AKT3 is one of the important molecules of the PI3K/AKT signaling pathway, we speculate that the signaling pathway might be involved in the mechanism of RUNX1. We preliminarily found that p-AKT level, which is positively related with the activation of the pathway, was also increased. Other researches confirmed that PI3K/AKT signaling pathway was closely associated with lung fibrosis in ARDS [18–20]. Hu C et al. found that inhibition of PI3K/AKT signaling pathway by wortmannin reduced TF expression in breast cancer MDA-MB-231 cells [21], and Simvastatin, an HMG-CoA reductase inhibitor, was found to inhibit thrombin-induced TF expression in human endothelial cells through inhibition of AKT [22]. All these research data suggested that PI3K/AKT pathway might be associated with alveolar hypercoagulation and fibrinolytic inhibition, and it is worth further to be investigated.

There are some limitations in our experiment. The relationship between RUNX1 and AKT3 was only testified in vitro but not in vivo. Meanwhile, we only observed the effect of RUNX1 overexpression on AKT3 but didn’t with RUNX1 down-regulation. Finally, we did not measure other indicators of hypercoagulation and fibrinolysis such as TAFI, APC, antithrombin and so on.

## Conclusion

Our results demonstrate that AKT3 is an important downstream target gene of RUNX1, through which RUNX1 regulates alveolar hypercoagulation and fibrinolytic inhibition in ARDS. RUNX1 / AKT3 pathway is expected to be a new target in exploring the pathogenesis of this syndrome.

### Supplementary Information

**Additional file 1: Fig. S1.** Transfection efficiency of si-AKT3 in AECII cells. The mRNA of AKT3 from the LPS-induced AECII which transfected with siRNA of AKT3. * P < 0.05, **P < 0.01. The values presented are the mean ± SD. (n = 3). Alveolar epithelial cells type II = AECII.

**Additional file 2: Table S1.** The RNA sequence for the complementary single‑stranded DNA. **Table S2.** The RNA sequence for the si-AKT3 gene and the sequence. **Table S3.** Sequence of primers in qPCR.

## Data Availability

The datasets used and/or analysed during the current study are available from the corresponding author on reasonable request. The CHIP-Seq read data have been deposited in the Gene Expression Omnibus database (GEO) under accession number GSE236748.

## Abbreviations

ARDS: Acute respiratory distress syndrome
RUNX1: Runt-related transcription factor 1
AECII: Alveolar epithelial cells type II
LPS: Lipopolysaccharide
BALF: Bronchoalveolar lavage fluid
WB: Western blots
qPCR: Quantitative real-time PCR
HE: Hematoxylin and eosin
DAD: Diffuse alveolar damage
LIS: Lung injury score
p-AKT: Phosphorylation-AKT
W/D ratio: Wet/dry ratio
