# Supplementary material for: RUNX1 targeting AKT3 promotes alveolar hypercoagulation and fibrinolytic inhibition in LPS induced ARDS
Source: Respir Res. 2024 Jan 24;25:54. doi: 10.1186/s12931-024-02689-2 (PMC10809548; doi:10.1186/s12931-024-02689-2)
Supplement: Supplementary file 1 — Additional file 1: Fig. S1. Transfection efficiency of si-AKT3 in AECII cells. The mRNA of AKT3 from the LPS-induced AECII which transfected with siRNA of AKT3. * P < 0.05, **P < 0.01. The values presented are the mean ± SD. (n = 3). Alveolar epithelial cells type II = AECII. [file 12931_2024_2689_MOESM1_ESM.docx]

**
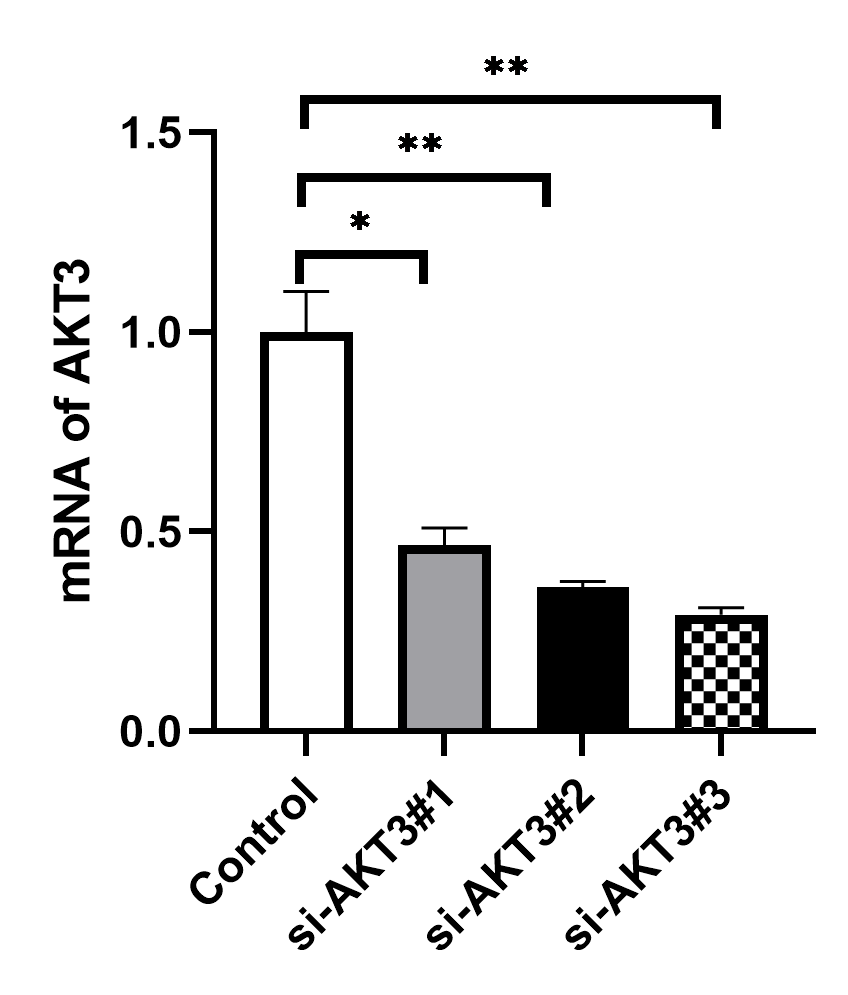
**

*Fig. S1. Transfection efficiency of si-AKT3 in AECII cells.* The mRNA of AKT3 from the LPS-induced AECII which transfected with siRNA of AKT3. * P＜0.05 , **P ＜0.01 and ****P ＜0.0001. The values presented are the mean ±SD. (n = 3). Alveolar epithelial cells type II=AECII.
