# Supplementary material for: RUNX1 targeting AKT3 promotes alveolar hypercoagulation and fibrinolytic inhibition in LPS induced ARDS
Source: Respir Res. 2024 Jan 24;25:54. doi: 10.1186/s12931-024-02689-2 (PMC10809548; doi:10.1186/s12931-024-02689-2)
Supplement: Supplementary file 2 — Additional file 2: Table S1. The RNA sequence for the complementary single‑stranded DNA. Table S2. The RNA sequence for the si-AKT3 gene and the sequence. Table S3. Sequence of primers in qPCR. [file 12931_2024_2689_MOESM2_ESM.doc]

**Addiitional Tables**

Table S1:The RNA sequence for the complementary single‑stranded DNA.

| RUNX1 | ATGCGTATCCCCGTAGATGCCAGCACGAGCCGCCGCTTCACGCCGCCTTCCACCGCGCTGAGCCCCGGCAAGATGAGCGAGGCGCTGCCGCTGGGCGCCCCGGATGGCGGCGCCGCCCTGGCCAGCAAGCTGAGGAGCGGCGACCGCAGCATGGTGGAGGTACTAGCTGACCACCCCGGCGAGCTAGTCCGCACCGACAGCCCCAACTTCCTCTGCTCCGTGCTGCCCACTCACTGGCGCTGCAACAAGACCCTGCCCATCGCTTTCAAGGTGGTAGCGCTGGGCGACGTTCCGGATGGCACTCTGGTCACCGTCATGGCGGGCAATGACGAAAACTACTCGGCGGAGCTGAGAAACGCCACCGCGGCCATGAAGAACCAAGTGGCGAGATTCAACGACCTCAGGTTTGTCGGTCGGAGCGGCAGAGGCAAGAGCTTCACTCTGACCATCACCGTCTTTACAAACCCCCCACAAGTTGCCACCTACCATAGAGCCATCAAAATCACAGTGGACGGCCCCCGAGAACCCCGAAGACATCGGCAGAAACTAGATGATCAGACCAAGCCCGGGAGTTTGTCCTTTTCCGAGCGGCTCAGTGAACTGGAGCAGCTGCGGCGCACGGCCATGAGGGTCAGCCCGCATCACCCAGCCCCCACGCCCAACCCTCGGGCCTCCTTGAACCACTCCACTGCCTTTAACCCTCAGCCTCAAAGTCAGATGCAGGATGCCAGGCAGATCCAGCCGTCCCCACCGTGGTCCTATGACCAGTCTTACCAGTACCTGGGGTCCATCACCTCTTCTGTGCACCCGGCGACACCCATTTCTCCCGGCCGAGCCAGTGGCATGACCAGCCTCTCAGCGGAACTTTCCAGTCGACTCTCAACAGCCCCGGACCTGACAGCCTTCGGCGACCCGCGCCAGTTCCCCACGCTGCCGTCCATCTCCGACCCGCGCATGCACTACCCAGGCGCCTTCACCTACTCGCCTCCTGTCACATCGGGCATCGGCATTGGCATGTCAGCCATGAGCTCGACCTCTCGCTACCACACCTACCTGCCGCCGCCCTACCCAGGCTCCTCGCAGGCGCAGGCCGGGCCCTTCCAGACCGGCTCGCCCTCCTACCACCTGTACTACGGCACCTCGGCAGGCTCCTACCAATTCTCCATGGTGGGCGGCGAGCGATCACCCCCGCGCATCCTGCCGCCCTGCACCAACGCGTCCACCGGAGCCGCGCTGCTCAACCCCAGCCTCCCCAGCCAGAGCGACGTGGTGGAAACCGAGGGCAGCCATAGCAACTCGCCCACCAACATGCCACCCGCGCGCCTGGAGGAGGCTGTCTGGCGGCCCTACtaa |
| --- | --- |
| NC | GTACCTCGCCTTATTTCTATCTTACGTCAAGAGCGTAAGATAGAAATAAGGCTTTTTGGAAA |

NC, negative control.

Table S2:The RNA sequence for the si-AKT3 gene and the sequence

| si-AKT3#1 | 5'-GGAUGCAUCUACAACCCAUTT ‑3' |
| --- | --- |
| si-AKT3#2 | 5'-GCUUUGGACUAUCUACAUUTT ‑3' |
| si-AKT3#3 | 5'-GCCUUUCUACAACCAGGAUTT ‑3' |

Table S3:Sequence of primers in qPCR

| **Ollgo Name** | **Sequence** |
| --- | --- |
| AKT3 forward | 5’-AATGATGTGTGGGAGGTTG-3’; |
| AKT3 reveres | 5’-GAGCCCTGAAAGCAATGA-3; |
| RUNX1 forward | 5’-CCTCAGCCTCAAAGTCAGATG-3’; |
| RUNX1 reveres | 5’-GGACCCCAGGTACTGGTAAGA-3’; |
| TF forward | 5’-ACTCAAGCACAGGAAAAAC-3’; |
| TF reveres | 5’-TGGAGAAAATCACGGCTTGTAC-3; |
| PAI-1 forward | 5’-GCCTGTTCCACAAGTCTGATG-3’; |
| PAI-1 reveres | 5’-ATGAACATGCTGAGGGTTTTCG-3’; |
| GAPDH forward | 5’-GACATGCCGCCTGGAGAAAC-3’; |
| GAPDH reveres | 5’-AGCCCAGGATGCCCTTTAGT-3’. |
